# Anthocyanidins Inhibit Growth and Chemosensitize Triple-Negative Breast Cancer via the NF-κB Signaling Pathway

**DOI:** 10.3390/cancers13246248

**Published:** 2021-12-13

**Authors:** Farrukh Aqil, Radha Munagala, Ashish K. Agrawal, Jeyaprakash Jeyabalan, Neha Tyagi, Shesh N. Rai, Ramesh C. Gupta

**Affiliations:** 1 UofL Health-Brown Cancer Center, 580 S. Preston St., Rm 304E, Baxter II Research Building, University of Louisville, Louisville, KY 40202, USA; farrukh.aqil@louisville.edu (F.A.); ashish.phe@iitbhu.ac.in (A.K.A.); jp3pbiotech@gmail.com (J.J.); neha.tyagi@tamu.edu (N.T.); 2 Department of Medicine, University of Louisville, Louisville, KY 40202, USA; 3 Department of Pharmacology and Toxicology, 580 S. Preston St., Rm 304E, Baxter II Research Building, University of Louisville, Louisville, KY 40202, USA; 4 Department of Biostatistics and Bioinformatics, University of Louisville, Louisville, KY 40202, USA; shesh.rai@louisville.edu

**Keywords:** breast cancer, anthocyanidins (Anthos), paclitaxel, chemosensitization, drug resistance, metastasis

## Abstract

**Simple Summary:**

Breast cancer is the most common female cancer diagnosed in the U.S. and the second most common cause of cancer death in women. Chemotherapeutics used to treat breast cancer often have side effects, which are sometimes life-threatening. Moreover, the tumors can develop resistance over time, making breast cancer treatment challenging. In this paper, we show that the oral administration of colored pigments isolated from bilberry/blueberry, called anthocyanidins (Anthos), significantly decrease MDA-MB-231 orthoxenograft tumor volume, inhibit the growth and metastasis of breast cancer, sensitize drug-resistant tumor cells, and exhibit a lower rate of lymph node and lung metastasis, compared to control. Our results also suggest regulation of cell-cycle progression and inhibition of NF-κB activation as mechanisms underpinning the anti-proliferative activity of Anthos in breast cancer. These mechanistic insights are expected to be valuable for clinical translation of berry Anthos, either alone or as adjuvant to chemotherapy, for the treatment of breast cancer patients.

**Abstract:**

Triple-negative breast cancer (TNBC) is an aggressive subtype of breast cancer. Due to the lack of drug-targetable receptors, chemotherapy is the only systemic treatment option. Although chemotherapeutic drugs respond initially in TNBC, many patients relapse and have a poor prognosis. Poor survival after metastatic relapse is largely attributed to the development of resistance to chemotherapeutic drugs. In this study, we show that bilberry-derived anthocyanidins (Anthos) can inhibit the growth and metastasis of TNBC and chemosensitize paclitaxel (PAC)-resistant TNBC cells by modulating the NF-κB signaling pathway, as well as metastatic and angiogenic mediators. Anthos administered orally significantly decreased MDA-MB-231 orthoxenograft tumor volume and led to lower rates of lymph node and lung metastasis, compared to control. Treatment of PAC-resistant MDA-MB-231Tx cells with Anthos and PAC in combination lowered the IC_50_ of PAC by nearly 20-fold. The combination treatment also significantly (*p* < 0.01) decreased the tumor volume in MDA-MB-231Tx orthoxenografts, compared to control. In contrast, Anthos and PAC alone were ineffective against MDA-MB-231Tx tumors. Our approach of using Anthos to inhibit the growth and metastasis of breast cancers, as well as to chemosensitize PAC-resistant TNBC, provides a highly promising and effective strategy for the management of TNBC.

## 1. Introduction

Breast cancer (BC) is the most common cancer in women, accounting for about 30% of female cancers, followed by lung cancer (13%), with approximately 3.5 million BC survivors in the United States alone [1]. Despite advances in early detection and treatment modalities, metastasis is a great challenge in BC patients [2]. Approximately 90% of BC deaths are attributed to metastatic breast cancer (MBC), with an overall survival rate of only 22% [3]. Unlike the significant progress made in the prognosis of estrogen-responsive breast cancers, the prognosis for triple-negative breast cancer (TNBC) remains dismal.

TNBC is characterized by its unique molecular profile, aggressive nature, distinct metastatic patterns, and lack of targeted therapies, and accounts for 10–17% of all BC [4]. TNBC is characterized by a lack of estrogen receptor (ER), progesterone receptor (PR), and HER2 expression, and is particularly associated with an aggressive pathology [5]. Moreover, 20–40% of all BCs metastasize within 3 to 10 years following the original diagnosis, and approximately 90% of BC deaths are attributed to metastasis, with an overall survival rate of only 16% [6]. At present, the mainstay of treatment for patients diagnosed with TNBC is cytotoxic chemotherapy. Not only are the adjuvant treatments used for MBC that target hormone receptors ineffective for TNBC, but they may also increase resistance to conventional therapies, posing a great challenge for treatment.

Signal transduction pathways have been often considered as potential targets in elucidating the molecular mechanisms of small molecules, including phytochemicals. A new prospect in research to elucidate the mechanisms of potential anti-cancer compounds is the recent discovery of molecular links between inflammation and cancer [2]. Nuclear factor-kappaB (NF-κB) is a ubiquitous redox-sensitive transcription factor that includes six family members and regulates a wide range of cellular events. It has been implicated in the pathogenesis of many inflammation-associated diseases. NF-κB binds to IκBα and is sequestered in the cytoplasm. Phosphorylation mediated by the IκB kinase (IKK) complex and successive ubiquitination degrades IκBα through the function of proteasomes. Due to their strong anti-inflammatory and antioxidant properties, Anthos have been proposed to demonstrate anti-cancer activity through the inhibition of NFκB-related pathways.

Resistance to chemotherapy is a major obstacle, and there are no standard practices for the effective management of MBC. The underlying biochemical and genetic causes of MBC resistance remain unclear. Over 80% of anti-tumor agents can be transported by ATP-binding cassette, which is frequently found to be elevated and may contribute to chemoresistance in BCs [7]. First-line chemotherapy provides only a 30–70% response rate; however, many patients show relapse and require additional chemotherapy within 6 to 10 months. Regrettably, the response rates remain only around 20–30% [8]. It is highly urgent that some unconventional new approaches be developed for the prevention and treatment of drug resistance and MBC. A user-friendly strategy is to use a combination of efficacious natural non-toxic compounds along with standard chemotherapeutic drugs.

Berries and berry bioactives have begun to receive significant attention recently, due to their much higher antioxidant activity when compared with other common fruits and vegetables. Reports have indicated blueberry Anthos [6] as possessing antioxidant, anti-inflammatory, anti-cancer, anti-diabetic, and cardio- and neuroprotective effects [9,10,11,12,13,14]. Several laboratories—including our own—have demonstrated their protective effect against carcinogen-induced cancers in animal models. A series of papers from the Stoner group initially showed that black raspberry and blackberry are effective against chemically induced colon [15] and esophageal [16] cancers. Preliminary results from a colon cancer clinical trial from the same groups have revealed 50% regression rates of rectal polyps [17]. Our series of studies have shown the significant inhibition of BC in an estrogen-sensitive ACI rat model by both blueberry and black raspberry [18,19,20], as well as for cigarette smoke-mediated lung cancer in a mouse model (our unpublished data), indicating that berries are chemopreventive beyond the GI tract. Berry bioactives also have significant therapeutic activity against lung [6], breast [21], ovarian [22], and colon cancer [23].

The chemopreventive and therapeutic activities of berries have been credited to the presence of colored pigments called anthocyanins. Berries in different parts of the world, such as blueberry in the U.S., bilberry in Europe, and jamun in Asia and South America, have been shown to contain glycosides of cyanidin (Cy), delphinidin (Dp), petunidin (Pt), peonidin (Pe), and malvidin (Mv). The sugar-free counterparts of anthocyanins are anthocyanidins. A compelling body of literature has suggested that anthocyanidins have various pharmacological properties, including the inhibition of cancer cell growth in vitro and in vivo, the induction of apoptosis, and so on. Our studies against lung cancer have suggested that anthocyanidins present a higher degree of efficacy than anthocyanins. Furthermore, we have also shown a significant synergistic activity when using a combination of anthocyanidins, such as a native mixture isolated from bilberry, compared to individual entities [6].

In this manuscript, we investigate the anti-cancer activity of bilberry-derived Anthos against TNBC using MDA-MB-231 and MDA-MB-236 BC cells, both in vitro and in vivo. We also demonstrate the chemosensitization of drug-resistant BC cells by the Anthos in combination with the chemo drug, PAC, and investigate the mechanism underlying the anti-cancer and anti-metastatic effects of the Anthos, with respect to the NF-κB signaling pathway, as well as metastatic and angiogenic mediators.

## 2. Materials and Methods

### 2.1. Materials

A native mixture of Anthos (>95% pure) was isolated from a standardized highly enriched extract of bilberry using solvent extractions and C18 chromatography [24]. Bilberry (a European ‘cousin’ of blueberry) contains essentially the same anthocyanidin profile as blueberry. MTT (3-[4,5-dimethylthiazol-2-yl]-2,5-diphenyltetrazolium bromide) was purchased from Alfa Aesar (Ward Hill, MA, USA). RIPA cell lysis buffer, BCA protein assay kit, PVDF membranes, phosphate-buffered saline (PBS), ECL chemiluminescence reagent, and Bolt 4–12% Bis-Tris phosphate gels were purchased from ThermoFisher (Rockford, IL, USA). Dulbecco’s Modified Eagle’s Medium (DMEM), L-15 media, penicillin, and streptomycin were purchased from Life Technologies (Gibco, Grand Islands, NY, USA). Fetal bovine serum (FBS) was purchased from ThermoFisher (Rockford, IL, USA). The human recombinant TGFβ-1 (#8915) and TNFα (#8902) were purchased from Cell Signaling Technology (Danvers, MA, USA).

### 2.2. Cell Culture

BC cells, MDA-MB-231, MDA-MB-436, and HCC1937 were purchased from American Type Culture Collection (ATCC, Manassas, VA, USA). L-15 media was used to grow MDA-MB-231 and MDA-MB-236 cells, while HCC1937 was grown in McCoy’s Medium supplemented with 10% fetal bovine serum and 1% antibiotics (penicillin/streptomycin). All cell lines were maintained at 37 °C in a humidified chamber at 5% CO_2_, except for MDA-MB-231 and MDA-MB-436, which were grown without CO_2_.

### 2.3. Anti-Proliferative Activity

The anti-proliferative activity of the Anthos against various cancer cells was assessed by MTT assay, as described previously [6]. IC_50_ values were calculated using the CalcuSyn software Version 2.0 (Biosoft, Cambridge, UK).

### 2.4. Flow Cytometry

For apoptosis assays, MDA-MB-231, MDA-MB-236, and HCC1937 cells were treated with different concentrations of Anthos for 24–72 h. Post-treatment, cells were collected and stained with annexin V-FITC and PI, according to the manufacturer’s guidelines (Invitrogen, Carlsbad, CA, USA). The cells were then evaluated for apoptosis by flow cytometry. The apoptotic cells were determined using a BD FACScan flow cytometer (Becton Dickinson, San Jose, CA, USA).

For cell cycle analysis, treated cells were fixed in ice-cold 70% ethanol for 30 min at 4 °C, centrifuged again, and washed twice with PBS. The cells were resuspended in 1 mL of 20 μg/mL PI and 100 μg/mL RNase A in PBS, then incubated for 30 min at room temperature. DNA content was analyzed using a BD FACScan flow cytometer (Becton Dickinson, San Jose, CA, USA). The population of cells in each cell cycle phase was determined using the FlowJo software v7.2.5 (Treestar, Ashland, OR, USA).

### 2.5. Wound Healing Assay

The wound healing assay was performed using 2-well inserts from ibidi^®^ (Munich, Germany) placed in 24-well plates, as described previously. Briefly, BC cells were suspended in culture media (100 cells/μL) and 100 μL of the cell suspension was added to each of the two wells in the cell inserts. The cells were incubated for 24 h to allow for cell attachment, and then cell-free gaps (wounds) 1 mm wide were created by removing the culture inserts. The wells were then treated with various concentrations of Anthos. The wound areas were monitored by light microscopy at 0, 24, and 48 h. At each of these time points, images of the wound areas were taken and quantified using the Wimasis Image Analysis software (WimScratch, Cordoba, Spain).

### 2.6. Trans-Well Migration and Invasion Assays

The trans-well migration and invasion assays were performed using uncoated and Matrigel-coated 8 µm pore size trans-well chambers (BD Bioscience, San Jose, CA, USA), respectively. BC cells were treated with Anthos (0–200 μM) in the presence or absence of TGFβ for 1 h. For the migration assay, 4 × 10^4^ cells were suspended in serum-free media and seeded into the top compartments of the trans-well chambers. Media supplemented with FBS as chemoattractant was added to the bottom chamber, to induce cell migration. After 24 h, the migrated cells were fixed in 4% paraformaldehyde, permeabilized using 100% methanol, and stained using 0.2% toluidine blue. The number of migrated cells was counted in five random fields under a microscope. In the invasion experiment, the same procedure was performed by seeding 80 × 10^4^ cells/well in Matrigel pre-coated trans-well chambers. Data are presented as representative microphotographs.

### 2.7. Electrophoretic Mobility Shift Assay

Analysis of NF-κB (DNA binding activity) was carried out through electrophoretic mobility shift assay (EMSA), as previously described [25]. The details are provided in the Supplementary Materials. DNA–protein complexes were visualized and quantified by Packard InstantImager (Packard Instruments, Downers Grove, IL, USA).

### 2.8. Protein Extraction and Western Blot Analysis

MDA-MB-231, MDA-MB-236, and HCC1937 cells were treated with various concentrations of Anthos for 24–72 h, and tumor tissues from an orthotopic breast cancer study were lysed in RIPA buffer containing protease inhibitor cocktail (Thermo Fisher Scientific, Waltham, MA, USA). Equal amounts of protein were separated by SDS-PAGE, and Western blot analysis was performed as described elsewhere [6,26]. Briefly, after transferring the proteins, the PVDF membrane was probed for the expression of specific proteins against various antibodies. The densities of each protein band, relative to the internal loading control (β-actin), were quantified using the ImageJ software (NIH, Bethesda, MD, USA).

### 2.9. Immunoprecipitation and IKKβ Kinase Activity Assay

First, we immunoprecipitated IKK complexes from cell lysates using anti-IKKγ antibodies (B-3) conjugated to agarose. Cell lysates (1–4 mg per sample) were incubated with antibodies (1–8 mg per sample) overnight at 4 °C under gentle rotation. Protein A Sepharose CL-4B beads (GE Healthcare Life Sciences; 10–30 µL per sample) were added to the tubes and rotated at 4 °C for 1 h. Beads were precipitated by centrifugation at 800× *g* for 30 s and washed three times with cold lysis buffer.

The in vitro IKKβ Kinase Activity assay was conducted with the harvested IKK complexes, using the Cyclex IKK Assay kit from MBL International (Woburn, MA, USA), following its instructions with no modifications.

Briefly, partially purified recombinant IKKβ and 10 µL of each sample or standard was added to the assay plate on ice. IKKβ-positive control (Cat# CY-E1176-2) was included in each assay, as a positive control for phosphorylation. Kinase reaction buffer (90 µL) was added to initiate the Kinase reaction and incubated for 30 min at 30 °C. The wells were washed four times with wash buffer (containing 2% Tween-20). Residual wash buffer was removed by gentle tapping. Next, 100 µL of Anti-phospho-IkBa serine32 monoclonal antibody (AS-2E8) was pipetted into each well and incubated for 30 min at RT. Wells were washed five times. Later, 100 µL of HRP-conjugated anti-mouse IgG was pipetted into each well and incubated for 30 min at RT. The wells were then washed five times again. Then, 100 µL of Substrate Reagent (containing chromogenic substrate, tetra-methylbenzidine; TMD) was added to each well and incubated for 5–15 min at RT. Finally, 100 µL of Stop Solution (containing 1NH2SO4) was added to each well. The absorbent signal in each well was measured with a spectrophotometric plate reader at dual 450/540 nm. All samples and standards were assayed in duplicate.

### 2.10. RT-PCR Analysis

Total RNA was isolated from cells using Trizol reagent (Invitrogen, Carlsbad, CA, USA). RNA samples (50 ng) were reverse transcribed and amplified using the Power SYBR Green RNA to CT 1-Step Kit according to the manufacturer’s guidelines (Applied Biosystems, Waltham, MA, USA). The relative mRNA expression levels of MDR-related molecules, such as MRP1, MDR1, MRCP, and MVP, normalized to GAPDH or β-actin, were determined. The fold change for each sample was determined using the 2^−^^ΔΔ^^Ct^ relative quantification method.

### 2.11. Animal Studies

The animal care and treatments were carried out in strict accordance with the protocol approved by the Institutional Animal Care and Use Committee (IACUC) of the University of Louisville.

A pilot study was launched to establish the orthotopic tumor xenografts of MDA-MB-231, MDA-MB-236, and HCC1937 cells, in order to identify the optimal model for anti-tumor activity studies. Five- to six-week old Athymic nude mice and NOD-Scid mice were randomized into 6 groups (*n* = 4). Exponentially growing cells were detached by trypsinization, washed, and re-suspended in serum-free media. Cell suspensions (2.5 × 10^6^) were mixed (at 1:1 volume) with Matrigel (BD Bioscience, Bedford, MA, USA) and injected under the second inguinal nipple on the right ventral side of the mammary pad. Animals were provided an AIN-93M purified diet and water ad libitum. Tumor volume, diet consumption, and animal health were monitored weekly. When tumors grew to 800 mm^3^, all animals were euthanized, and blood and tumor tissues were collected. To determine whether the orthotopic tumor xenografts resulted in distant metastasis, a gross examination of lymph nodes was conducted at the end of the study. Lymph nodes were collected at the time of euthanasia, and their size was calculated to examine the metastasis. Only MDA-MB-231 showed exponential growth of tumors and was also found to be highly metastatic; hence, they were chosen for the subsequent anti-tumor activity study.

For the anti-tumor activity study, 2.5 × 10^6^ MDA-MB-231 cells were injected under the second inguinal nipple on the right ventral side of the mammary pad, as described above. When the tumor grew to 120 mm^3^, animals were divided into three groups (*n* = 10–12) and treated with Anthos. Group 1 received vehicle control, whereas Groups 2 and 3 received the Anthos at 30 mg/kg and 60 mg/kg, respectively, by oral gavage three times a week. When tumor volume in the control group reached 800 mm^3^, all animals were euthanized. Blood and selected tissues were collected. At euthanasia, we examined for gross metastasis of lymph nodes and collected lymph nodes, lung, liver, and spleen for histopathological examination of metastasis. During the gross examination, lymph nodes received a score of 1+, 2+, or 3+, depending on the size of the enlarged lymph nodes 0.1–0.5 mm, 0.5–1 mm, and >1 mm, respectively; non-detectability of lymph node enlargement was scored as 0.

In a separate study, the chemosensitization potential of Anthos was tested. Taxol-resistant TNBC MDA-MB-231Tx cells (5 × 10^6^ cells) were injected under the second inguinal nipple on the right ventral side of the mammary pad. When the tumors reached approximately 120 mm^3^, animals were randomized into four groups. Group 1 received vehicle control, whereas Group 2 received Anthos at 60 mg/kg by oral gavage three times a week. Group 3 received PAC at 4 mg/kg i.p. once a week, and Group 4 received a combination of Anthos and PAC at the same doses as in the individual groups. Animal health, tumor growth, and metastasis were monitored as described above.

### 2.12. Statistical Analysis

Statistical analysis was performed using the GraphPad Prism statistical software (version 4.03; La Jolla, CA, USA). Statistical significance of differences in various parameters was evaluated by unpaired Student’s *t*-tests. Data in the xenograft studies are expressed as mean ± standard error of the mean (SEM) (*n* = 10). A value of *p* < 0.05 was considered statistically significant.

## 3. Results

### 3.1. Anthos Inhibit the Growth of TNBC Cells by Modulating Cell-Cycle Regulatory and Survival Proteins

**Figure 1:**
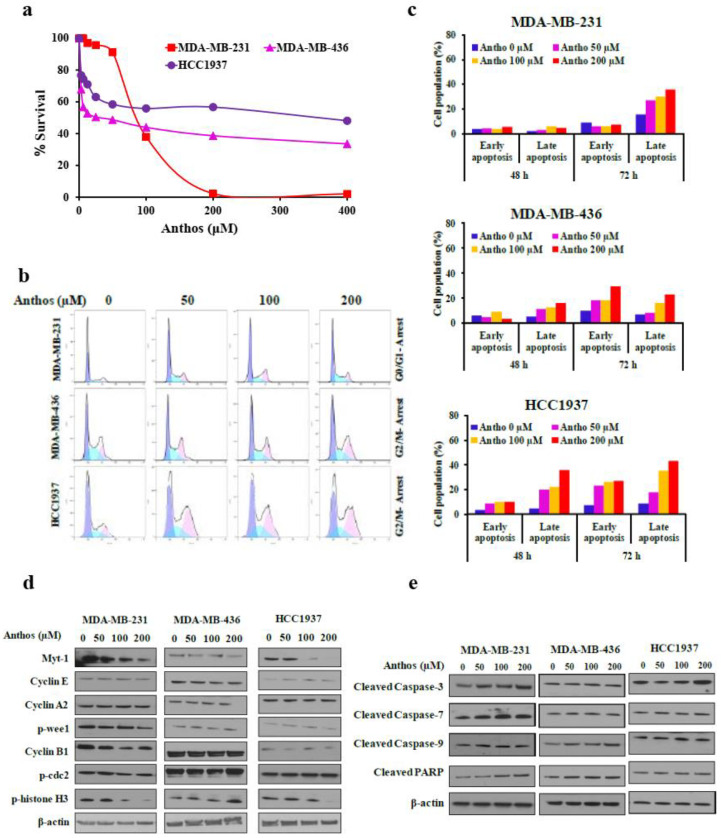
Effect of Anthos on cell viability, cell-cycle progression, apoptosis, and cell-cycle regulatory proteins: (**a**) BC cells were treated with a native mixture of Anthos (0–400 μM) isolated from bilberry extract for 72 h and MTT assay was performed. Data denote the mean of three experiments (SD < 10%); (**b**,**c**) MDA-MB-231, MDA-MB-436, and HCC1937 cells were treated with Anthos (0–200 μM), and cell-cycle arrest (**b**) and apoptosis (**c**) were determined after staining cells with propidium iodide by flow cytometry; (**d**,**e**) BC cells were treated with Anthos (0–200 μM) for 48 h and cell lysates were analyzed by Western blot for indicated proteins. Equal loading was confirmed by β-actin. Densitometry data are presented in Figures S2 and S3.

We determined the anti-proliferative activity of Anthos against three TNBC cell lines—MDA-MB-231, MDA-MB-436, and HCC1937—by MTT assay. Anthos showed dose-dependent activity against all tested cell lines. The IC_50_ values, in ascending order, were 100 ± 0.7, 240 ± 0.5, and >400 µM against MDA-MB-231, MDA-MB-436, and HCC1937, respectively. This indicated that MDA-MB-231 cells were more sensitive to Anthos, with several-fold lower IC_50_ compared to MDA-MB-436 and HCC1937 cells (see Figure 1a). Interestingly, Anthos did not affect the growth of normal epithelial keratinocyte (HEK) cells, despite the high concentrations tested (400 µM; Figure S1).

The effect of Anthos on cell-cycle progression was assessed through flow cytometry by PI staining of TNBC cells after treatment with Anthos in a dose- and time-dependent manner. Our results indicated significant G0/G1 arrest in MDA-MB-231 cells, and G2/M arrest in MDA-MB-436 and HCC1937 cells (Figure 1b and Figure S4). These observations also occurred dose-dependently, with modulation of several key cell-cycle regulatory kinases and protein targets after Anthos treatment, including Cyclin A, Cyclin B1, Cyclin E2, p-CDC2, p-wee1, p-histone H2, and Myt1 (Figure 1c).

We next examined the induction of apoptosis by Anthos in TNBC cell lines through flow cytometry using Annexin V and propidium iodide (PI) staining. As shown in Figure 1d and Figure S13, a dose- and time-dependent increase in the percentage of early and late apoptotic cells after treatment with Anthos was noted. These findings confirm apoptosis-mediated cell killing by Anthos. Examination of cell survival- and apoptosis-related proteins indicated a dose-dependent progressive increase in the levels of cleaved caspase-3, caspase-7, and caspase-9 proteins in response to Anthos treatment. These trends were more visible in MDA-MB-231 cells, compared to MDA-MB-436 and HCC1937. An increase in cleaved PARP level with Anthos further confirmed apoptosis in these cells (Figure 1e). These results suggest that Anthos caused apoptosis by the activation of caspases in TNBCs.

### 3.2. Anthos Prevent NF-κB Activation and IκB Kinase Activity

**Figure 2:**
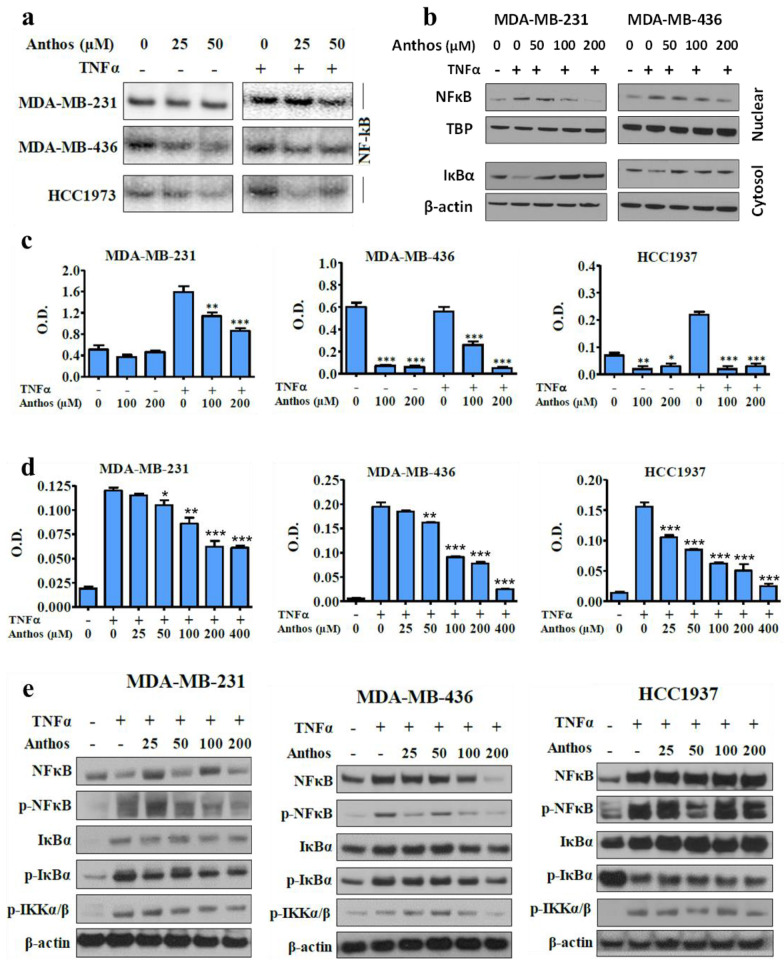
Effect of Anthos on NF-κB activation, nuclear translocation, and related protein molecules in TNBC: (**a**) BC cells were treated with Anthos (0–50 μM) in the presence or absence of TNFα for 48 h and nuclear extracts were analyzed by EMSA; (**b**) Cells were treated with Anthos (0–200 μM) for 48 h, and nuclear and cytosol lysates were probed for NF-κB and IκBα proteins by Western blot. Equal loading was confirmed by tata binding protein (TBP) and β-actin, respectively; (**c**) Effect of bilberry Anthos on NF-κB activation. Breast cancer cells were treated with Anthos (100 and 200 μM) in presence or absence of TNFα for 48 h, and nuclear extracts were analyzed by ELISA: (**d**) Whole-cell lysates were prepared and immune-precipitated with anti-IKKβ antibody. The immuno-complex kinase assay was performed by ELISA using a CycLex^®^ IKKα and β Kinase Assay/Inhibitor Screening Kit; (**e**) Whole-cell lysates were probed for IκBα and P65 levels. Equal loading confirmed by β-actin. Statistical analysis was carried out using student’s *t*-test; * *p* < 0.05; ** *p* < 0.01; *** *p* < 0.001. Densitometry data are presented in Figures S5–S7.

We investigated the ability of the Anthos to inhibit constitutive and TNFα-induced NF-κB activation and NF-κB binding activity by gel mobility shift assay (EMSA). In MDA-MB-436 and HCC1937 cells, Anthos at 50 µM diminished both constitutive and TNFα-induced NF-κB activation, indicating anti-inflammatory activity. However, such inhibition in MDA-MB-231 cells was much less effective (Figure 2a). Further examination of NF-κB binding activity by ELISA in the presence of Anthos indicated effective inhibition of TNFα-induced NF-κB binding activity in all TNBC cell lines tested. While Anthos inhibited uninduced NF-κB binding activity in MDA-MB-436 and HCC1937, it did not affect MDA-MB-231 cells (Figure 2b). These observations were in agreement with our EMSA assay findings.

To evaluate whether Anthos affect the nuclear translocation of NF-κB through IκB phosphorylation and/or degradation, we examined the status of IκB phosphorylation and degradation after treatment with TNFα in MDA-MD-231 and MDA-MB-436 cells. We observed phosphorylation after 10 min of treatment, followed by its degradation with time. MDA-MB-231 cells showed rapid degradation, compared to MDA-MB-436 cells (Figure S8). Based on this finding, cells were treated with TNFα for 10 min for the detection of IκB phosphorylation in subsequent experiments. Our results indicated increased cytoplasmic IκB (Figure 2c) with a decrease in nuclear NF-κB levels, in a dose-dependent manner. These results suggest that Anthos antagonize the nuclear translocation of NF-κB, inhibiting the phosphorylation of IκB and its subsequent degradation. Next, we determined whether Anthos target the cellular IKK complex for NF-κB inhibition by monitoring the kinase activity of IKK upon treatment with Anthos. BC cells were pretreated with increasing doses of Anthos for 1 h, followed by a 10 min challenge with TNFα. Cell lysates were subjected to immunoprecipitation with an IKKβ antibody. The kinase activity of immuno-precipitated IKK complexes was determined using the ELISA method. Treatment of Anthos resulted in the neutralization of IKK complex activity in all of the TNBC cells. The effect was more pronounced in MDA-MB-436 and HCC1937 cells, compared to MDA-MB-231 cells (Figure 2d). These results confirmed that the inhibitory action of the Anthos was due to its effect on the phosphorylation and degradation of IkBα, considering the phosphorylated levels of IkBα protein determined by immunoblot analysis. Anthos exhibited a strong ability to inhibit IkBα and NF-κB phosphorylation in a concentration-dependent manner (Figure 2e and Figure S13).

### 3.3. Anthos Modulate EMT Markers to Inhibit Metastasis

**Figure 3:**
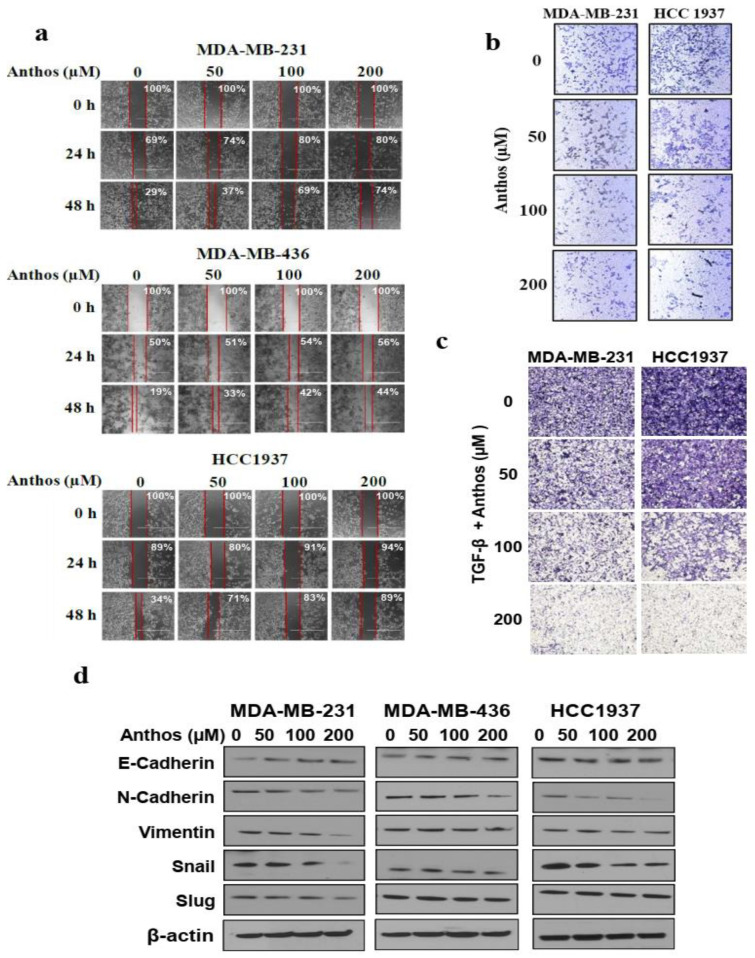
Effect of Anthos on cell migration, invasion, and EMT pathway proteins: (**a**) BC cells seeded in 2-Well Culture-Insert in a 24-well plate (Ibidi^®^, Munich, Germany), resulting in a 1 mm wide wound. The inserts were removed after overnight incubation and cells were treated with Anthos (0–200 μM). Migration was assessed after 24 h and 48 h of Anthos treatment, represented as the percentage reduction in wound area; (**b**,**c**) Cells seeded in matrigel-coated transwell inserts. The bottom chamber was treated with Anthos (0–200 μM) in the absence (**b**) and presence (**c**) of TGFβ. Invasive cells at the bottom of the trans-well insert were visualized after staining with 0.2% toluidine blue; (**d**) Cells were treated with Anthos (0–200 μM) for 48 h and whole-cell lysates were probed for EMT pathway proteins. Equal loading confirmed by β-actin. Densitometry data from panel D are presented in Figure S9.

The effect of Anthos on the metastasis of TNBC cells in vitro was assessed through wound-healing and trans-well migration and invasion studies. Anthos at 100 and 200 µM resulted in greater abrogation of metastatic potential of MDA-MB-231 and HCC1937 cells up to 48 h (Figure 3d), while MDA-MB-436 cell migration ability was the least affected by Anthos (Figure 3a). MDA-MB-231 and MDA-MB-436 cells were the most aggressive among the TNBC cell lines tested. Anthos treatment was less effective in inhibiting the migration ability of MDA-MB-436 cells, compared to MDA-MB-231 cells. Further, the effect of Anthos on the invasive behavior of TNBC cells was tested using Matrigel trans-well migration and invasion assay. Our results indicated that Anthos could effectively inhibit both native (Figure 3b) and TGFβ -induced invasiveness of MDA-MB-231 and HCC1973 (Figure 3b).

Next, we investigated the effects of the Anthos on EMT markers, in order to validate the reduced migration and invasion abilities observed above. Our findings indicated that Anthos induced E-cadherin and inhibited N-cadherin, Vimentin, Snail, and Slug expression and, consequently, the metastatic phenotype TNBC cells. These findings demonstrate, for the first time, the role of Anthos in the regulation of EMT through the induction of E-cadherin and inhibition of Snail (Figure 3c).

### 3.4. Anthos Inhibited Growth and Metastasis of Orthoxenograft Tumors

Before examining the effect of the Anthos on orthotopic TNBC xenografts in a mouse model, we first launched a pilot study to establish the growth rate of TNBC xenografts in our laboratory. The three TNBC cell lines were tested for six weeks, in order to establish tumor growth rates and metastatic ability in two mouse models; namely, athymic nude and NOD-Scid mice. HCC1937 and MDA-MB-231 cells exhibited linear growth rates in the NOD-Scid mice, while MDA-MB-436 cells showed linear growth of orthotopic tumors in athymic nude mice. Although MDA-MB-231and HCC1937 xenografts were well-established in nude mice, the variability among tumor size was larger, compared to those established in NOD-Scid mice. MDA-MB-436 cells exhibited larger variability in the NOD-Scid mouse model (Figure S10). To determine if the orthotopic tumor xenografts resulted in distant metastasis, we conducted a gross examination of lymph nodes at the end of the study. Our findings indicated enlargement of lymph nodes with the highly metastatic MDA-MB-231 and MDA-MB-436, while HCC1937 cells showed weak metastasis in NOD-Scid mice. None of the cell lines resulted in metastasis in athymic nude mice.

**Figure 4:**
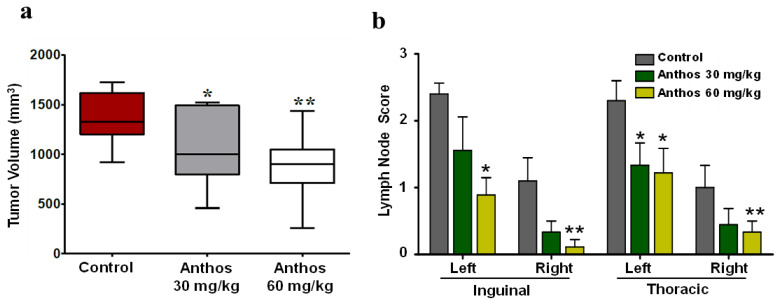
Effect of Anthos on tumor growth inhibition and lymph node metastasis: (**a**) NOD-Scid mice were inoculated (Ventral right, under second inguinal nipple) with MDA-MB-231 BC cells (2.5 × 10^6^ cells) to produce an orthotopic tumor. When the tumor grew to ~120 mm^3^, animals were treated (oral gavage, three times a week) with Anthos (30 mg/kg or 60 mg/kg b.wt.). Control groups received PBS; (**b**) At euthanasia, lymph nodes were scored for size (as 0, 1+, 2+, or 3+). Data represent average ± SE (*n* = 9–10). Data represent average ± SE (*n* = 9–10). Statistical analysis was carried out using student’s *t*-test; * *p* < 0.05; ** *p* < 0.005.

Given the above findings, we chose to use MDA-MB-231 to test Anthos, as it exhibited less variability in tumor size and more linear growth and metastasis, compared to the other cell lines, in the NOD-Scid mouse model. In this study, 2.5 × 10^6^ MDA-MB-231 cells were injected under the second inguinal nipple on the right ventral side of the mammary pad. When the tumor volume reached approximately 120 mm^3^, the animals were randomized into three groups. Group 1 received vehicle control, whereas Groups 2 and 3 received Anthos at 30 mg/kg and 60 mg/kg, respectively, by oral gavage three times a week. After 6 weeks of intervention, both doses of Anthos resulted in a significant decrease in tumor volume, compared to control. However, the anti-tumor effect at the higher dose was more pronounced (Figure 4a). At euthanasia, we examined for gross metastasis in lymph nodes and collected lymph nodes, lung, liver, and spleen for histopathological examination of metastasis. During the gross examination, lymph nodes received a score of 1+, 2+, and 3+, depending on the size of the enlarged lymph nodes: 0.1–0.5 mm, 0.5–1 mm, and >1 mm, respectively; absence of lymph node enlargement was scored as 0. Results of the observation of thoracic and inguinal lymph nodes are shown in Figure 4b and Table S1. Our findings suggest that macro-metastasis of lymph nodes was significantly decreased by Anthos intervention at both doses, compared with the vehicle control group. The higher dose of Anthos (60 mg/kg) led to smaller tumor size and a lower total number of enlarged lymph nodes, compared to both the lower dose and the control group. Histopathological examination confirmed lymph node and lung metastasis of MDA-MB-231 cells. No metastasis for the liver or spleen was observed in any of the groups. High-dose Anthos exhibited a lower rate of lymph nodes and lung metastasis, compared to the lower dose Anthos.

### 3.5. Anthos Chemosensitize PAC-Resistant Cells by Modulating Drug Transporters

**Figure 5:**
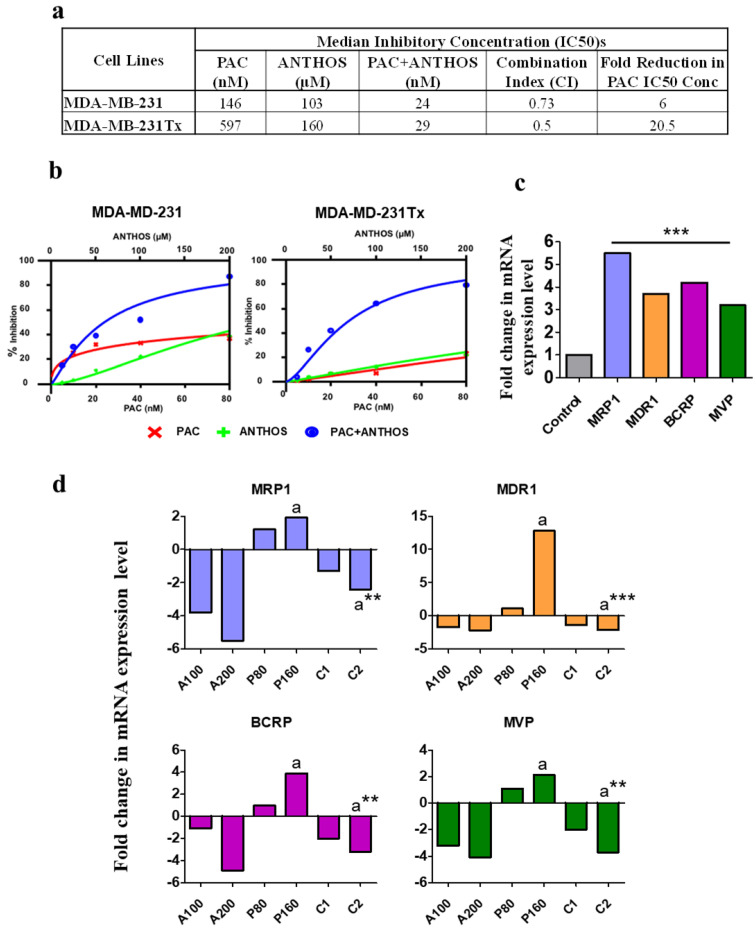
Anti-proliferative and synergistic activity of Anthos against parental and paclitaxel (PAC)-resistant BC cells and effects on resistant gene markers: (**a**,**b**) Drug-sensitive (MDA-MB-231) and drug-resistant (MDA-MB-231Tx) cells were treated with Anthos and PAC, alone and in combination, at various concentrations. Anti-proliferative activity was determined by MTT assay. IC_50_ values, of individual and combination treatments, and combination index were calculated using the Calcusyn software; (**c**) Expression levels of resistant gene markers (MRP1, MDR1, BRCP, and MVP) were determined using RT-PCR. The bar graph depicts the fold increase in mRNA expression of resistance proteins in MDA-MB-231Tx cells compared to parental MDA-MB- 231 cells; (**d**) MDA-MB-231Tx were treated with indicated doses of Anthos (A; μM) and PAC (P; nM). C1 and C2 indicate combination treatment with PAC:Anthos at 1:1250; C1 = PAC 80 nM + Anthos 100 μM while C2 = PAC 160 nM + Anthos 200 μM. Significant differences between groups are indicated by a and a**; ** *p* < 0.01, *** *p* < 0.001.

After establishing the anti-cancer effects of Anthos against MDA-MB-231 cells and tumors, we sought to investigate whether Anthos had an inhibitory effect on PAC-resistant MDA-MB-231 (MDA-MB-231Tx) cells. The effect of the Anthos alone and in combination with PAC on the cell growth inhibition of parental MDA-MB-231 and PAC-resistant MDA-MB-231Tx cells was determined by MTT assay. Our findings indicated significant inhibition of cell proliferation of MDA-MB-231Tx cells (Figure 5a), and that the IC_50_ values of Anthos and PAC alone were 160 µM and 597 nM, respectively; however, the IC_50_ value of PAC was substantially reduced (29 nM) when PAC was combined with Anthos. Thus, Anthos–PAC combined treatment resulted in a near 20-fold lowering of the IC_50_ of PAC (Figure 5a).

As it is well-known that drug transporters play a crucial role in cancer drug resistance [27], we examined the expression levels of several drug transporters, including ABCB1 (MDR1), ABCC1 (MRP1), ABCG2/BCRP, and LRP/MVP, in MDA-MB-231Tx cells by RT-PCR. Our findings indicated over 3.2- to 5.5-fold increased expression of these drug transporters, compared to parental MDA-MB-231 cells (Figure 5b). Later, we examined whether the chemosensitizing effects of Anthos were mediated by the modulation of drug-transporter proteins. To this end, MDA-MB-231Tx cells were treated with Anthos, PAC, and PAC + Anthos (Figure 5d). While treatment of MDA-MB-231Tx cells with PAC resulted in a further increase in the expression of all of the resistance-conferring drug transporter proteins, treatment with Anthos exhibited dose-dependent down-regulation of these makers. Furthermore, combination treatment with PAC + Anthos showed the reversal of resistance marker expression levels (Figure 5c).

### 3.6. Anthos Inhibits PAC-Induced NF-κB to Overcome Drug Resistance

**Figure 6:**
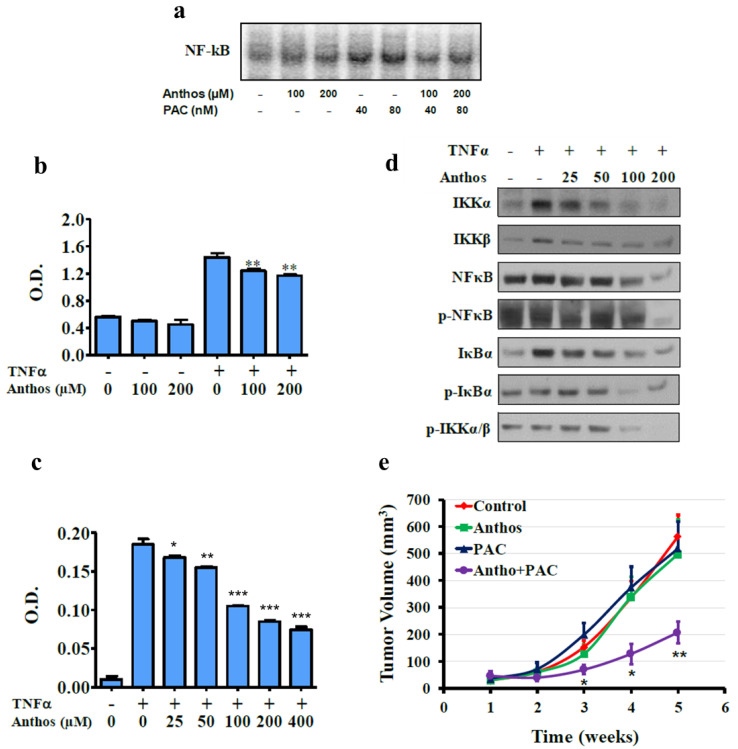
Effect of Anthos on NF-κB activation, NF-κB pathway proteins, and tumor growth inhibition of PAC-resistant BC cells: (**a**) MDA-MB-231Tx cells were treated with Anthos and PAC, alone and in combination, for 48 h and nuclear extracts were analyzed by EMSA; (**b**) BC cells were treated with Anthos (100 and 200 μM) in presence or absence of TNF-α for 48 h and nuclear extracts were analyzed by ELISA; (**c**) Whole-cell lysates were prepared and immune-precipitated with anti-IKKβ antibody. The immuno-complex kinase assay was performed by ELISA using CycLex^®^ IKKα and β Kinase Assay/Inhibitor Screening Kit; (**d**) BC cells were treated with Anthos (0–200 μM) for 48 h. Whole-cell lysates were probed for IκBα and P65 levels. Equal loading confirmed by β-actin; (**e**) Effect of Anthos on MDA-MB-231Tx tumor growth inhibition. NOD-Scid mice were inoculated with MDA-MB-231Tx BC cells (2.5 × 10^6^ cells) under the nipple to produce orthotopic tumors. When tumor xenografts grew to ~120 mm^3^, animals were treated with Anthos (60 mg/kg b. wt.; 3 times/week; oral gavage), PAC (4 mg/kg, b. wt; once weekly; i.p.), or both. Control groups were treated with PBS. Data represent average ± SE (*n* = 10). Statistical analysis was c using student’s *t*-test; * *p* < 0.05; ** *p* < 0.01, *** *p* < 0.001. Densitometry data from panels A and D are presented in Figure S12.

NF-κB is well-known to be constitutively activated in many types of cancer, including TNBC cells [28]. Moreover, PAC is known to induce NF-κB activation [29]. We investigated the ability of Anthos, alone and in combination with PAC, to inhibit NF-κB activation in MDA-MB-231Tx cells. Our findings suggest that combination treatment inhibited PAC-induced NF-κB activity, while Anthos alone did not affect it (Figure 6a). Further, NF-κB binding activity in presence of Anthos indicated effective inhibition of TNFα-induced NF-κB binding activity in MDA-MB-231Tx, compared to constitutive levels (Figure 6b). We also examined the status of IκB phosphorylation and degradation after treatment with TNFα in MDA-MB-231Tx. We observed that similar to MDA-MB-231 cells, MDA-MB-231Tx cells exhibited IκBα phosphorylation after 10 min of TNFα treatment, followed by its degradation with time (Figure S11). Pre-treatment with Anthos followed by a 10 min challenge with TNFα resulted in the neutralization of IKK complex activity in MDA-MB-231Tx cells (Figure 6c).

Next, we evaluated the effect of Anthos on IKKα, IKKβ, NF-κB, p-NF-κB, IkBα, p-IkBα, and p-IKKα/β in MDA-MB-231Tx cells. Anthos decreased the TNFα-induced levels of pNF-κB and p-IkBα (Figure 6d). As phosphorylation of IkBα is mediated by IKKs [30], we also examined whether stimulation of IkBα by Anthos was mediated by IKKα and IKKβ. Figure 6d shows that Anthos treatment, indeed, decreased the protein levels of IKKα, IKKβ, and p-IKKα/β, indicating the ability of Anthos to facilitate apoptosis by impeding the NF-κB survival pathway as a consequence of IkBα stimulation.

Finally, we tested the effect of Anthos and PAC, alone and in combination, on the growth and metastasis of MDA-MB-231Tx orthoxenografts in NOD-Scid mice. In this study, 5 × 10^6^ MDA-MB-231Tx cells were injected under the second inguinal nipple on the right ventral side of the mammary pad. When the tumors reached approximately 120 mm^3^ in volume, the animals were randomized into four groups. Group 1 received vehicle control, whereas Group 2 received the Anthos at 60 mg/kg by oral gavage three times a week, Group 3 received PAC at 4 mg/kg (i.p.) once a week, and Group 4 received a combination of Anthos and PAC at the same respective doses as used individually. After 5 weeks of intervention, both Anthos and PAC alone were found to be ineffective in decreasing tumor volume. However, the combination group resulted in a significant decrease in tumor volume, compared to the control, as early as 3 weeks (Figure 6e). The gross examination revealed the absence of lymph nodes or distant metastasis. Histopathological examination confirmed the absence of metastasis in any of the groups.

## 4. Discussion

BC treatment has been significantly improved due to advancements in surgery, radiotherapy, and adjuvant chemotherapy. Further, with the development of targeted therapies, the outcomes for patients diagnosed with hormone receptor-positive (HR+) and/or human epidermal growth factor receptor 2-positive (HER2+) BC has continued to improve. However, the same cannot be said for those affected with TNBC [31]. TNBC is extremely aggressive, more likely to metastasize than other subtypes of BC, and has a poor prognosis, compared with hormone-responsive sub-types [5]. In this study, we demonstrated that Anthos inhibited the growth of human TNBC cells, both in vitro and in vivo, by modulating cell-cycle regulatory and survival proteins, as well as by preventing NF-κB activation and IκB kinase activity. A direct effect of Anthos, in modulating EMT markers to inhibit metastasis and in chemosensitizing PAC-resistant cells by modulating drug transporters, was further demonstrated. We also showed, for the first time, that Anthos inhibits PAC-induced NF-κB to overcome drug resistance.

We, and others, have previously demonstrated the strong anti-oxidant activities of anthocyanins and Anthos [32,33]. Anthos scavenge free radicals and, thus, reduce damage to the genome of normal cells by oxidative stress and the subsequent malignant transformation by gene mutation, thereby preventing the occurrence of tumors [34]. In this study, we showed the strong anti-proliferative activity of Anthos against various TNBC cells, with the highest activity against MDA-MB-231. Anti-proliferative and anti-cancer activities have been previously shown, by both our group and others, against lung, breast, and ovarian cancer cells [6,22,33]. We have also demonstrated the higher activity of bilberry Anthos when used as a native mixture rather than individual entities [6]. MDA-MB-231 BC cells were more sensitive to Anthos, compared to other cell lines. The effect of Anthos on MDA-MB-231 was associated with G0/G1 arrest, while G2/M arrest was observed in MDA-MB-436 and HCC1937 cells. In agreement with other studies [35,36], we demonstrated that Anthos inhibited cell growth and cell-cycle progression in a dose-dependent manner. Cell-cycle arrest was also associated with the modulation of key protein targets, such as Cyclin A, Cyclin B1, Cyclin E2, p-CDC2, p-wee1, and p-histone H2. Cyclin B1 is involved in neoplastic transformation and, thus, promotes the proliferation of tumor cells. Therefore, its down-regulation—consequently reducing the activity of Cdk1/Cyclin B1—could block the aggressive proliferation of tumor cells [37].

A direct link between apoptosis and the cell cycle is supported by the fact that mitosis and apoptosis display very similar morphological features [38]. We noted that Anthos exhibits cell growth inhibition through the induction of apoptosis, resulting from the activation of Poly(ADP-ribose) polymerase (PARP) protein cleavage. These results are in agreement with other studies, in which Anthos and one of its constituents—delphinidin—has also been shown to cause apoptosis and PARP cleavage [35,36,39,40]. PARP activation takes place in response to DNA fragmentation during the induction of apoptosis and is, therefore, considered an important biomarker. It has been also reported that Anthos could induce apoptosis through the internal mitochondrial and external death receptor pathway [41].

NF-κB is known to be constitutively activated in several TNBC cells, including MDA-MB-231. NF-κB overexpression has been implied in aggressive tumor biology in BC, leading to poor prognosis; therefore, NF-κB positive tumors need to be treated aggressively. NF-κB activation has been correlated with high-grade, large tumor size, ER negativity, PR negativity, and HER-2/neu positivity in BC patients [42]. Anthos has been shown to act as NF-kB inhibitors by preventing the activation of IkB kinase B (IKKβ), IkB phosphorylation and degradation, and NF-kB/DNA binding and gene transcription [39]. We investigated the ability of Anthos to inhibit constitutive levels and TNFα-induced NF-κB activation by measuring NF-κB binding activity. Our results indicated a dose-dependent inhibition of TNFα-induced NF-κB binding activity in MDA-MB-231 and MDA-MB-436 cells, additionally confirming that Anthos exerted its effect through the phosphorylation and degradation of IkBα.

In tumors, NF-κB signaling is activated by TNFα, which stimulates cell survival, proliferation, epithelial–mesenchymal transition (EMT), migration, and chemo-resistance [43,44]. EMT in BC requires NF-κB activity [45], which induces and maintains EMT in model systems through two mechanisms: Up-regulation of EMT master-switch transcription factors and stabilization of Snail [46]. In the first mechanism of EMT, epithelial cells lose the epithelial markers E-cadherin and cytokeratin and acquire mesenchymal markers, including N-cadherin, fibronectin, vimentin, and some soluble metalloproteinases [47]. Our data showed that Anthos induced E-cadherin and inhibited N-cadherin, vimentin, snail, and slug expression, indicating the ability of Anthos to regulate EMT signaling through induction of E-cadherin and inhibition of Snail. Individual anthocyanidins (delphinidin, cyanidin, pelargonidin, petunidin, and malvidin) have been shown to alter the expression of snail [47]. Similar findings, in which anthocyanins modulated genes involved in EMT processes by inhibiting NF-κB, have been reported [48,49].

To determine the effect of Anthos on tumor growth and lymph node metastasis, MDA-MB-231 orthoxenografts were established in a NOD-Scid mouse model. The orthotopic xenograft models represent a clinically relevant model, with respect to the tumor’s primary site, micro-environment, and metastasis. Anthos significantly decreased tumor volume, compared to control; however, as expected, the effect was more pronounced at the higher dose (60 mg/kg). After anti-tumor efficacy, we examined the effect on metastasis and found that macro-metastasis of lymph nodes was significantly decreased by the Anthos intervention, compared with vehicle treatment. EMT is often activated during cancer invasion and metastasis. As Anthos inhibit molecules involved in EMT and downregulate various cyclins, including Cyclin B1, these could comprise a possible mechanism for Anthos to inhibit cancer metastasis. Our group, and others, have demonstrated the anti-cancer activity of Anthos in animal models [18,19,20,50]; however, this is probably the first report of Anthos against orthotopic BC.

Chemotherapeutic drugs, such as PAC, alone or in combination with anthracycline agents, are the first-line chemo drugs used for MBC treatment [51,52]. Resistance to chemotherapy, developed following the exposure of tumor cells to a chemotherapeutic agent, is believed to cause treatment failure in over 90% of MBC patients. Although patients generally have a favorable initial response to taxane regimens, the rapid development of resistance to taxanes is prevalent [52]. This prompted us to examine whether Anthos could chemosensitize resistant BC cells. Our data indicate that Anthos enhanced the efficacy of PAC by reducing its IC_50_ value, thus suggesting its chemosensitizing ability against PAC-induced drug resistance in MDA-MB-231Tx cells.

After induction of resistance to a single class of anti-cancer agents, tumor cells may subsequently exhibit resistance to other structurally unrelated drugs, which is referred to as “cross-resistance” or “multidrug resistance” (MDR). Overexpression of drug transporter proteins, such as PgP, MRP1, and BCRP has been reported to play a major role in MDR. Additionally, activation of the NF-κB signaling pathway is a common phenomenon in several PAC-resistant cancers, including TNBCs [53]. In this report, we observed multiple targets of Anthos to overcome acquired resistance to PAC in BC cells; first, by inhibition of NF-κB signaling molecules and, second, by reversal of MDR resistance markers expression levels. Anthos also phosphorylates TNFα-induced IκBα, followed by its degradation, suggesting a role of Anthos in modulating the NF-κB signaling pathway. Moreover, Anthos treatment decreased the protein levels of IKKα, IKKβ, and p-IKKα/β. As the phosphorylation of IKKα and IKKβ is associated with IKKs [30], these data suggest that Anthos cause apoptosis by stimulating IkBα, leading to NF-κB survival pathway obstruction. Further, Anthos-induced suppression of NF-κB activity could potentiate the effect of PAC, as observed by a synergistic effect under their combination. Similar activity has also been reported with another polyphenol, resveratrol, which inhibits TPA-induced phosphorylation of IκBα and subsequent p65 nuclear translocation in mouse skin by blocking IKKα and IKKβ [54].

We investigated the chemosensitizing potential of an Anthos and PAC combination to inhibit the growth and metastasis of PAC-resistant MBC orthoxenografts. PAC combined with Anthos showed strong anti-tumor efficacy. However, we noted the lack of effectiveness of treatment with Anthos alone in this model. This was somewhat expected, due to the aggressiveness of the tumor growth rate of MDA-MB-231Tx orthoxenografts. A similar finding of synergistic activity with Anthos has been observed against a lung cancer tumor xenograft [6]. These data confirm that the Anthos–PAC combination is effective and can synergistically inhibit the growth of drug-resistant BC.

## 5. Conclusions

In this report, we provided mechanistic insights into the mode of action of berry Anthos leading to the growth inhibition and apoptosis of TNBC cells. The bilberry bioactives (Anthos) inhibited the growth and metastasis of BC and chemosensitized the cells to the standard chemo drug, PAC, serving as an effective strategy for the management of metastatic TNBC. We gained mechanistic insights by examining multiple targets associated with cell proliferation, apoptosis, inflammation, invasion, and metastasis. For the first time, we report the significant inhibition of orthotopic TNBC (MDA-MB-231) xenograft tumor growth and lymph node metastasis through the oral administration of berry Anthos. Furthermore, our findings indicated the chemosensitizing ability of the Anthos against PAC-resistant BC cells, by inhibiting the expression of drug transporter markers. A synergistic effect between PAC and the Anthos was observed, with significant tumor growth reduction (>60%) in a PAC-resistant MDA-MB-231Tx xenograft. The effectiveness of Anthos will likely increase when delivered in milk exosomal formulations, as has been demonstrated against lung cancer [26] and ovarian cancer [22]. Together, these tumor inhibition and mechanistic insights will be valuable for clinical translation of Anthos, either alone or as adjuvant to chemotherapy, for the treatment of BC patients.

## Data Availability

The data presented in this study are available on request from the corresponding author. The data are not publicly available due to privacy.

## Supplementary Materials

The following are available online at https://www.mdpi.com/article/10.3390/cancers13246248/s1, Table S1: Gross examination of macro-metastasis following treatment with Anthos in an orthotopic model of TNBC, Figure S1: Effect of Anthocyanidins on normal epithelial keratinocytes cells, Figure S2: Densitometry data of blots in Figure 1a, Figure S3: Densitometry data of blots in Figure 1e, Figure S4: Effect of Anthos on cell-cycle progression in TNBC cells, Figure S5: Densitometry data of blots in Figure 2a, Figure S6: Densitometry data of blots in Figure 2b, Figure S7: Densitometry data of blots in Figure 2e, Figure S8: Anthos inhibits phosphorylation of IkBa dose-dependently in TNFa-induced TNBC cells, Figure S9: Densitometry data of blots in Figure 3d, Figure S10: Development of orthotopic models on TNBC in Athymic nude and NOD-skid mice, Figure S11: Anthos inhibits phosphorylation of IkBa dose-dependently in drug-resistant MDA-MB-231 cells, Figure S12: Densitometry data of blots in Figure 6d, Figure S13: Original Western Blot of Figure 1d, Figure 2e and Figure 3d.
