# Supplementary material for: Anthocyanidins Inhibit Growth and Chemosensitize Triple-Negative Breast Cancer via the NF-κB Signaling Pathway
Source: Cancers (Basel). 2021 Dec 13;13(24):6248. doi: 10.3390/cancers13246248 (PMC8699375; doi:10.3390/cancers13246248)
Supplement: Supplementary file 1 [file cancers-13-06248-s001.zip › cancers-1469756-supplementary.pdf]

cancers

Supplementary Materials: Anthocyanidins Inhibit Growth and Chemosensitize Triple-Negative Breast Cancer via the NF-κB Signaling Pathway

Farrukh Aqil, Radha Munagala, Ashish Agrawal, Jeyaprakash Jeyabalan, Neha Tyagi, Shesh N. Rai and Ramesh C. Gupta

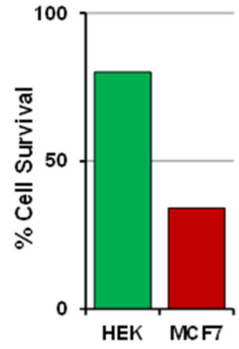

Figure S1. Effect of Anthocyanidins on cancer versus normal cells. Anti-proliferative activity of Anthos (400 µM) against normal epithelial keratinocytes (HEK) and the MCF-7 BC cells (C) as measured by MTT assay.

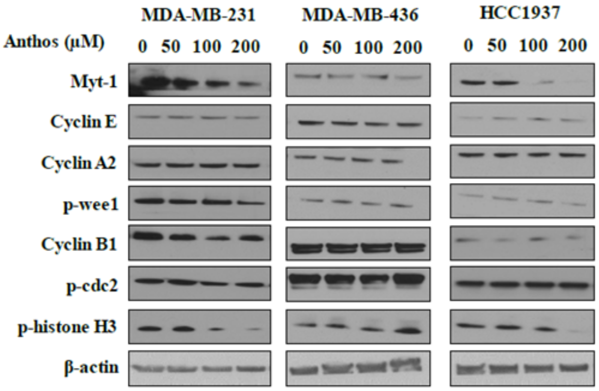

MDA-MB-231

Proteins	Relative Fold Change			
	Anthos 0	Anthos 50	Anthos 100	Anthos 200
Myt-1	1.0	0.48	0.29	0.13
Cyclin-E	1.0	1.14	1.26	1.00
Cyclin-A2	1.0	1.11	1.20	1.00
P-Wee1	1.0	0.81	1.00	0.59
Cyclin B1	1.0	0.88	0.79	0.80
p-cdc2	1.0	1.09	0.74	0.83
p-Histon H3	1.0	0.96	0.36	0.19

MDA-MB-436

Proteins	Relative Fold Change			
	Anthos 0	Anthos 50	Anthos 100	Anthos 200
Myt-1	1.0	0.63	0.76	0.42
Cyclin-E	1.0	0.75	0.59	0.64
Cyclin-A2	1.0	0.85	1.02	0.84
P-Wee1	1.0	1.20	0.87	1.09
Cyclin B1	1.0	0.77	0.72	0.66
p-cdc2	1.0	0.75	0.64	0.75
p-Histon H3	1.0	1.17	1.02	1.88

HCC1937

Proteins	Relative Fold Change			
	Anthos 0	Anthos 50	Anthos 100	Anthos 200
Myt-1	1.0	0.92	0.15	0.05
Cyclin-E	1.0	1.52	1.97	1.75
Cyclin-A2	1.0	0.93	0.88	1.07
P-Wee1	1.0	1.47	1.64	1.38
Cyclin B1	1.0	0.68	0.91	0.86
p-cdc2	1.0	1.06	1.16	1.21
p-Histon H3	1.0	0.98	0.75	0.15

Figure S2: Densitometry data of blots in Figure 1a.

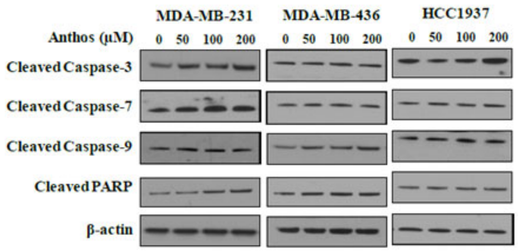

MDA-MB-231

Proteins	Relative Fold Change			
	Anthos 0	Anthos 50	Anthos 100	Anthos 200
Cleaved Capase-3	1.0	1.26	1.32	1.51
Cleaved Capase-7	1.0	1.43	2.27	1.41
Cleaved Capase-9	1.0	1.34	1.42	0.98
Cleaved PARP	1.0	1.12	1.87	1.99

MDA-MB-436

Proteins	Relative Fold Change			
	Anthos 0	Anthos 50	Anthos 100	Anthos 200
Cleaved Capase-3	1.0	0.95	0.89	0.85
Cleaved Capase-7	1.0	0.95	0.94	0.80
Cleaved Capase-9	1.0	1.38	1.16	1.58
Cleaved PARP	1.0	1.40	1.34	1.25

HCC1937

Proteins	Relative Fold Change			
	Anthos 0	Anthos 50	Anthos 100	Anthos 200
Cleaved Capase-3	1.0	0.84	1.02	1.48
Cleaved Capase-7	1.0	1.10	0.99	1.15
Cleaved Capase-9	1.0	1.24	1.51	1.31
Cleaved PARP	1.0	1.15	1.24	1.36

Figure S3: Densitometry data of blots in Figure 1e.

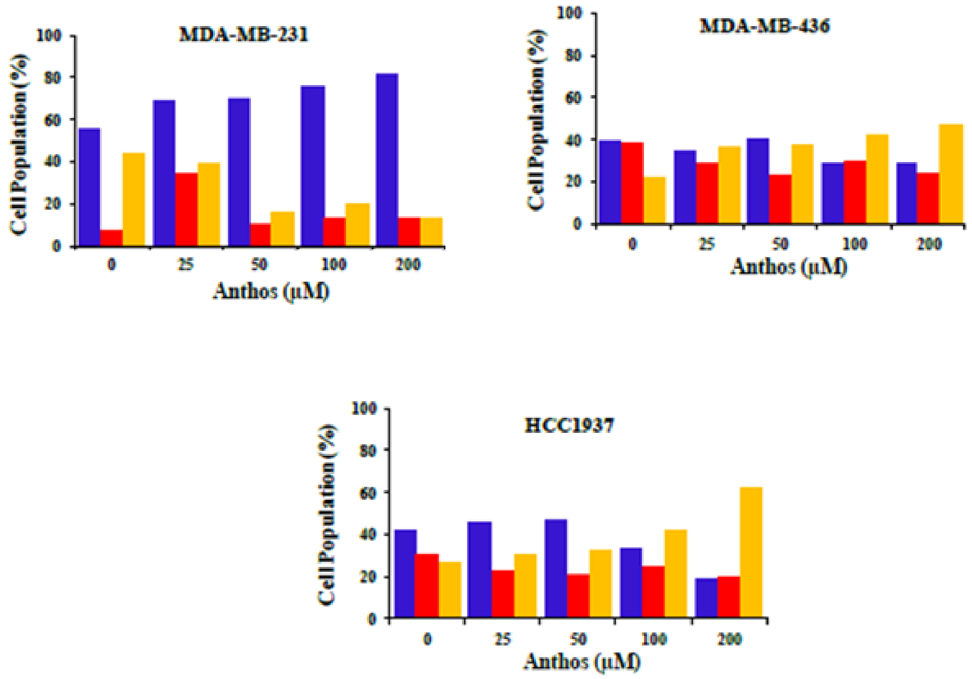

Figure S4. Effect of Anthos on cell cycle progression. MDA-MB-231, MDA-MB-436 and HCC1937 cells were treated with Anthos (0-200 μM) and cell cycle arrest was determined by flow cytometry after staining cells with propidium iodide.

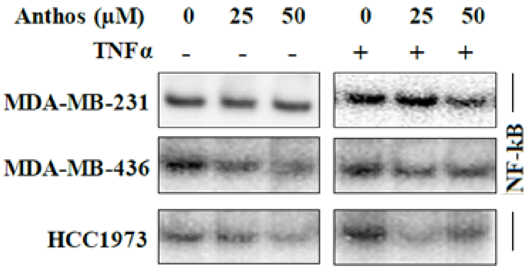

NF-kB

Cell lines	Relative levels		
	Anthos 0	Anthos 25	Anthos 50
MDA-MB-231	1.00	0.96	0.89
MDA-MB-436	1.00	0.69	0.44
HCC1973	1.00	0.84	0.40

Cell lines	Relative levels		
	Anthos 0	Anthos 25	Anthos 50
MDA-MB-231	1.00	0.96	0.72
MDA-MB-436	1.00	0.69	0.70
HCC1973	1.00	0.32	0.43

Figure S5: Densitometry data of blots in Figure 2a.

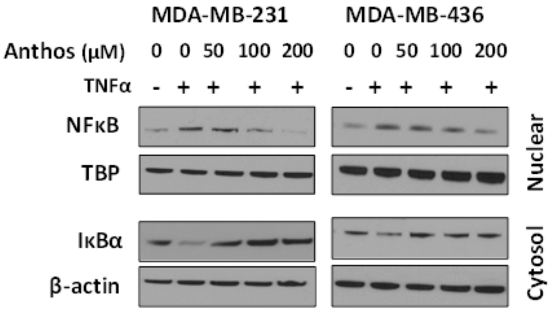

Treatment	Proteins	Relative levels				
Anthos (µM)		0	0	50	100	200
TNF		–	+	+	+	+
MDA-MB-231	NFkB	1.00	2.20	2.34	1.77	0.85
	IkBa	1.00	0.63	1.31	1.00	0.84
MDA-MB-436	NFkB	1.00	2.92	2.86	1.49	0.30
	IkBa	1.00	0.33	1.12	1.95	1.44

Figure S6: Densitometry data of blots in Figure 2b.

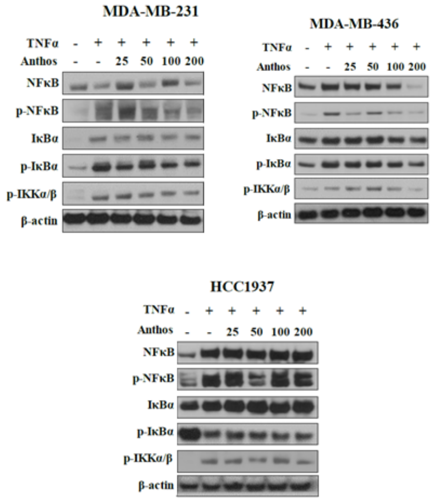

MDA-MB-231

Proteins	Relative levels					
	Anthos 0	TNF + Anthos 0	TNF + Anthos 25	TNF + Anthos 50	TNF + Anthos 100	TNF + Anthos 100
NFkB	1.00	0.57	1.65	0.78	1.28	0.86
p-NFkB	1.00	2.51	3.36	2.34	2.50	2.17
IKBα	1.00	2.18	2.45	2.53	2.09	2.02
p-IKBα	1.00	2.62	2.42	2.51	2.58	1.88
p-IKKα/β	1.00	1.68	2.08	1.51	1.88	1.77

MDA-MB-436

Proteins	Relative levels					
	Anthos 0	TNF + Anthos 0	TNF + Anthos 25	TNF + Anthos 50	TNF + Anthos 100	TNF + Anthos 100
NFkB	1.00	1.42	1.09	0.83	0.69	0.30
p-NFkB	1.00	2.46	1.28	1.40	1.04	0.99
IKBα	1.00	1.23	1.02	0.86	0.67	0.70
p-IKBα	1.00	2.02	1.47	1.25	1.13	0.78
p-IKKα/β	1.00	1.47	1.80	1.71	1.13	0.57

HCC1937

Proteins	Relative levels					
	Anthos 0	TNF + Anthos 0	TNF + Anthos 25	TNF + Anthos 50	TNF + Anthos 100	TNF + Anthos 100
NFkB	1.00	3.30	2.79	2.91	3.54	3.42
p-NFkB	1.00	1.94	1.57	1.06	1.92	1.71
IKBα	1.00	1.25	1.09	1.29	1.27	1.35
p-IKBα	1.00	0.45	0.38	0.36	0.35	0.31
p-IKKα/β	1.00	5.68	4.15	3.77	5.55	3.65

Figure S7: Densitometry data of blots in Figure 2e.

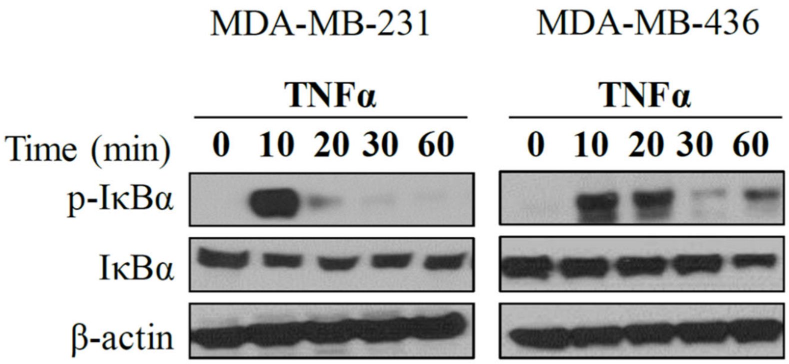

Figure S8. Effect of TNFα-induced phosphorylation and degradation of IκBα. Breast cancer cells MDAMB-231 and MDA-MB-436 were challenged with TNFα for 0-60 min. Whole cell lysates were probed for IκBα and p-IκBα proteins. Equal loading confirmed by β-actin.

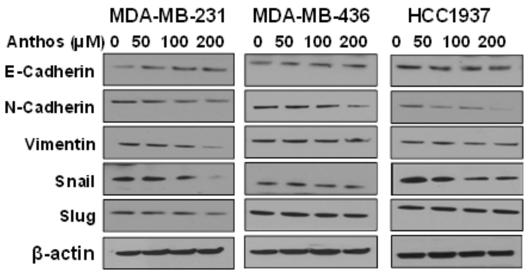

MDA-MB-231

Proteins	Relative Fold Change			
	Anthos 0	Anthos 50	Anthos 100	Anthos 200
E-Cadherin	1.00	1.55	2.25	2.08
N-Cadherin	1.00	0.54	0.45	0.37
Vim entin	1.00	0.61	0.51	0.15
Snail	1.00	0.63	0.40	0.06
Slug	1.00	0.55	0.60	0.33

MDA-MB-436

Proteins	Relative Fold Change			
	Anthos 0	Anthos 50	Anthos 100	Anthos 200
E-Cadherin	1.00	0.76	0.80	1.14
N-Cadherin	1.00	0.83	0.54	0.35
Vim entin	1.00	0.95	0.62	0.80
Snail	1.00	0.94	0.60	0.66
Slug	1.00	0.98	0.64	0.83

HCC1937

Proteins	Relative Fold Change			
	Anthos 0	Anthos 50	Anthos 100	Anthos 200
E-Cadherin	1.00	0.77	0.99	0.99
N-Cadherin	1.00	0.44	0.67	0.26
Vim entin	1.00	0.99	0.84	0.98
Snail	1.00	0.64	0.59	0.61
Slug	1.00	0.94	0.96	1.01

Figure S9: Densitometry data of blots in Figure 3d.

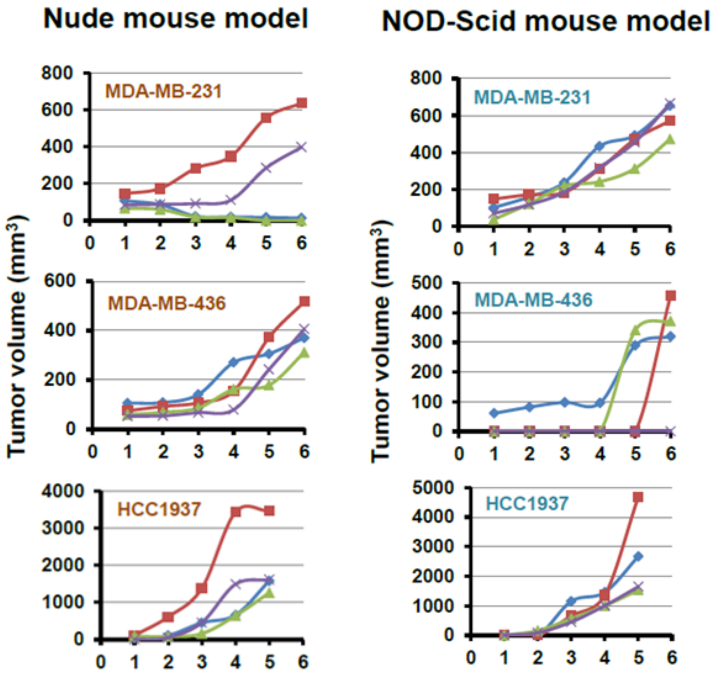

Figure S10. Pilot animal study establishing tumor growth rates. Athymic nude and NOD-Scid mice were inoculated with breast cancer cells (2.5 x 106 cells) under the nipple to produce orthotopic tumor. The tumor growth rate was monitored for 6 weeks.

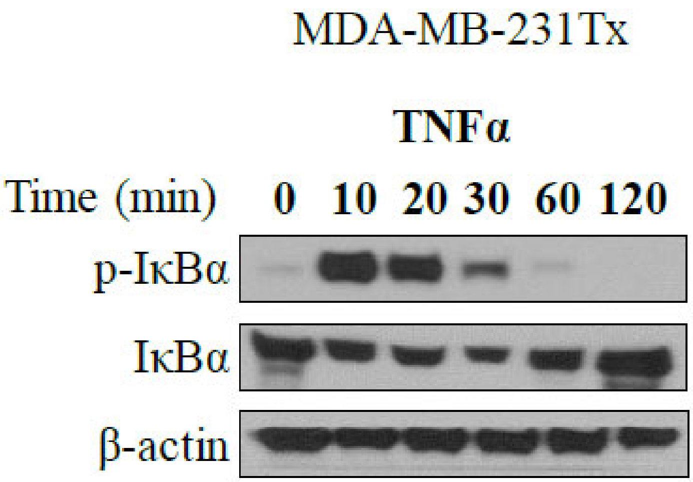

Figure S11. Effect of TNFα-induced phosphorylation and degradation of IκBα. Drug resistant BC cells MDAMB-231Tx were challenged with TNFα for 0-60 min. Whole cell lysates were probed for IκBα and p-IκBα proteins. Equal loading confirmed by β-actin.

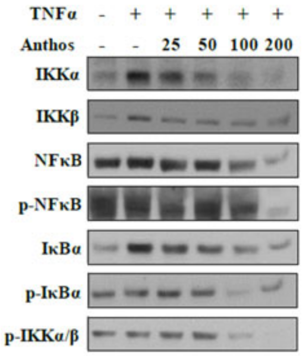

Proteins	Relative levels					
	Anthos 0	TNF + Anthos 0	TNF + Anthos 25	TNF + Anthos 50	TNF + Anthos 100	TNF + Anthos 100
Ikkα	1.00	2.60	0.88	0.55	0.35	0.16
Ikkβ	1.00	3.42	0.62	0.60	0.62	0.67
NFkB	1.00	1.42	0.89	0.87	0.59	0.22
p-NFkB	1.00	0.99	0.89	0.88	0.87	0.08
IkBα	1.00	3.41	0.77	0.60	0.38	0.22
p-IkBα	1.00	1.19	1.15	0.96	0.31	0.49
p-IkBα/β	1.00	1.06	1.08	1.01	0.41	0.17

Figure S12: Densitometry data of blots in Figure 6d.

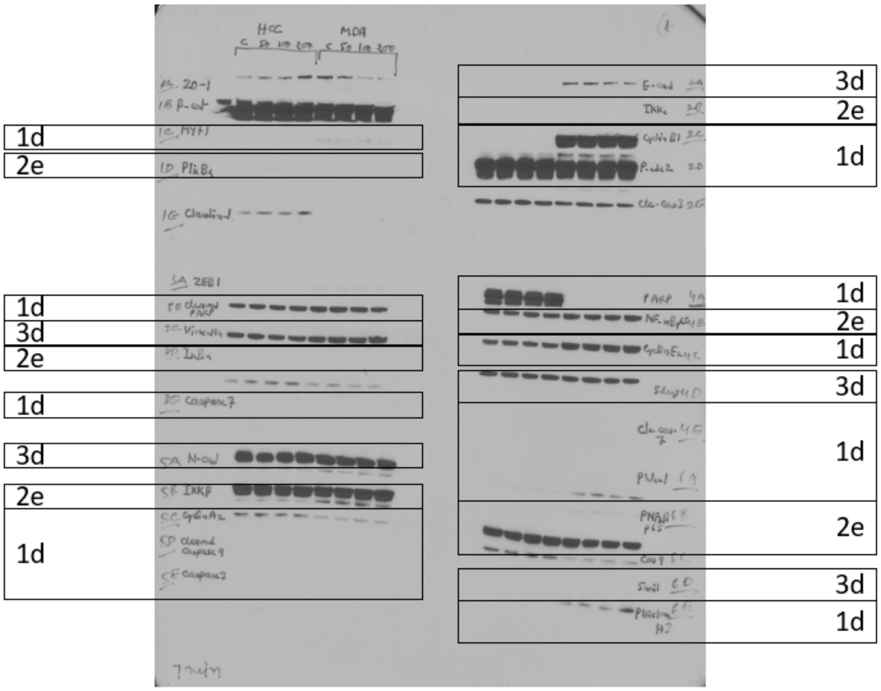

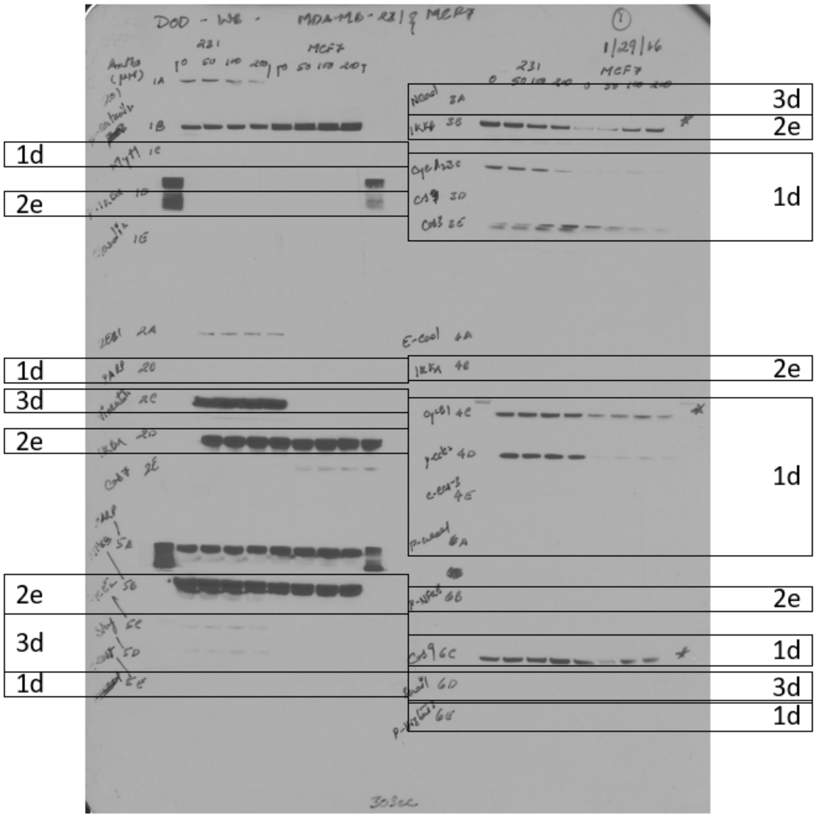

3d
2e
1d
1d
2e
1d
2e
3d
2e
1d
2e
2e
3d
1d
1d
3d
1d

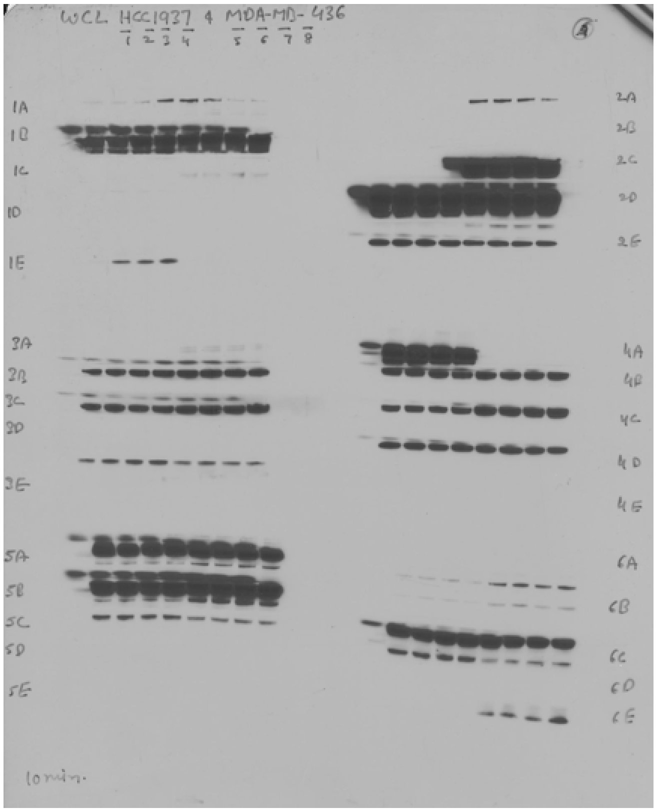

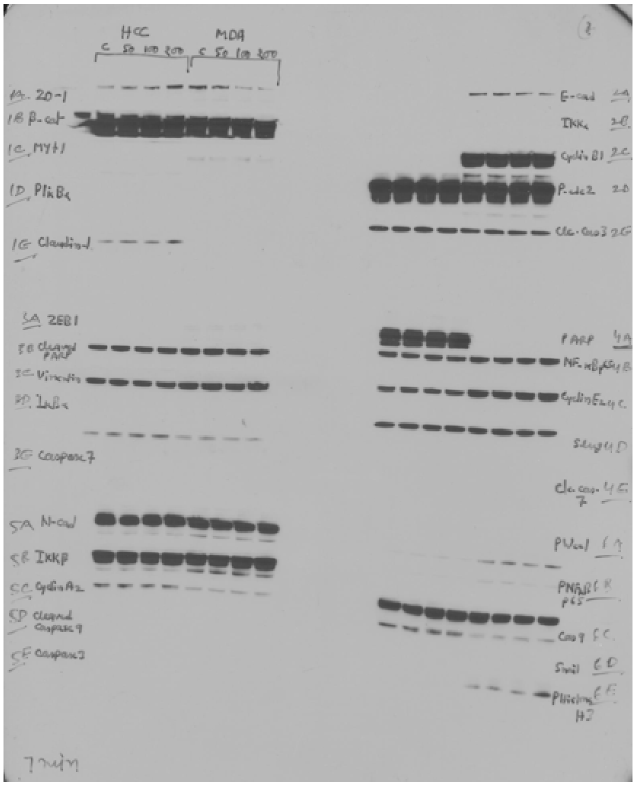
HCC
MDA
7 min

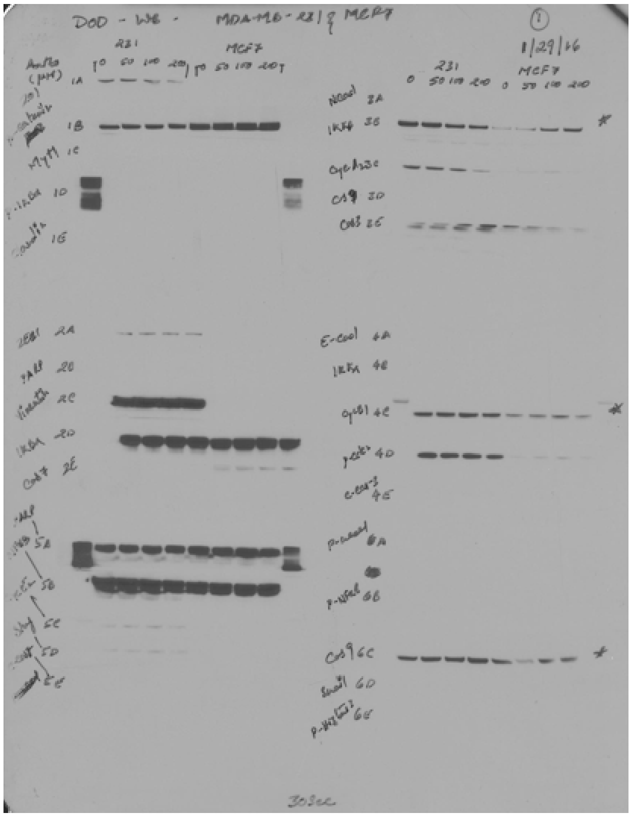
DOD - WB - MDA-MB-231 & MCF7
1/29/16
231
MCF7
30 sec

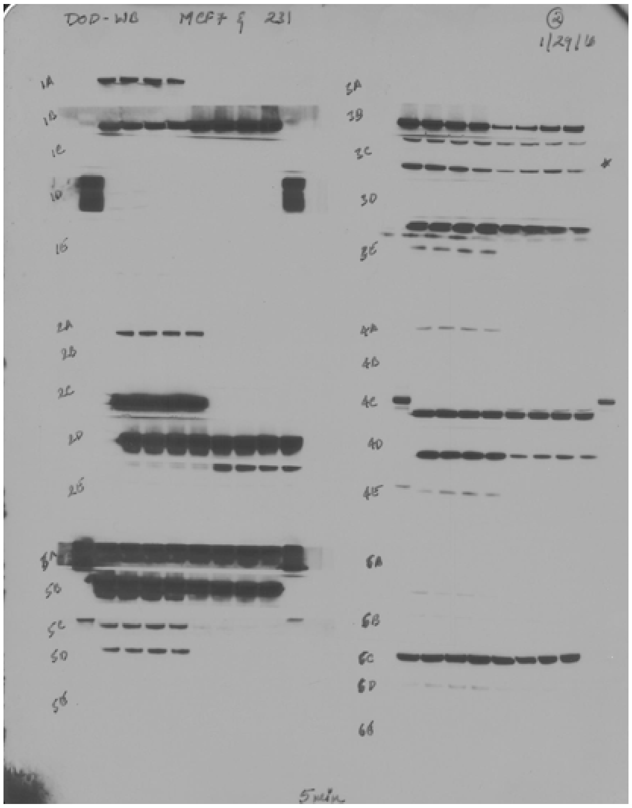
DOD-WB MCF7 & 231
1/29/16
5 min

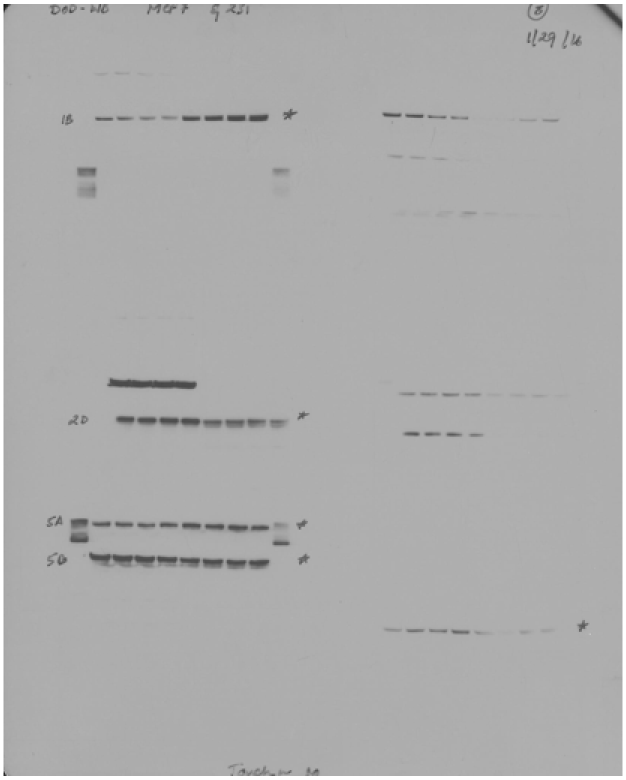
DOD-WB MCF7 & 231
1/29/16
1B
2D
5A
5B

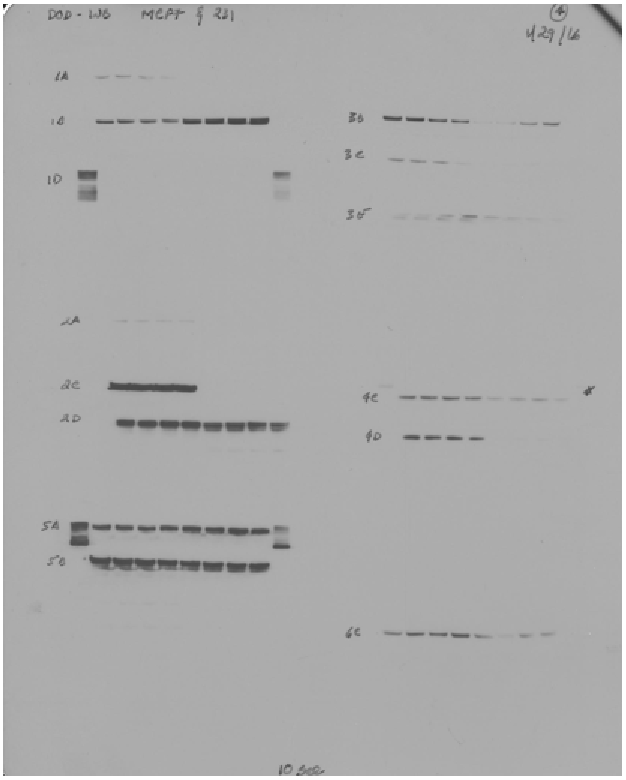
DOD-WB MCF7 & 231
1/29/16
1A
1B
1D
2A
2C
2D
5A
5B
3B
3C
3E
4C
4D
6C
10 sec

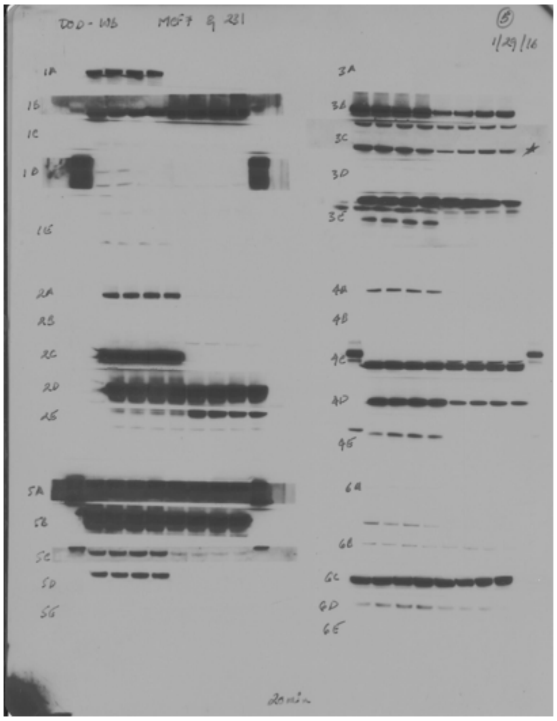
DOD-WB MCF7 & 231
1/29/16
20 min

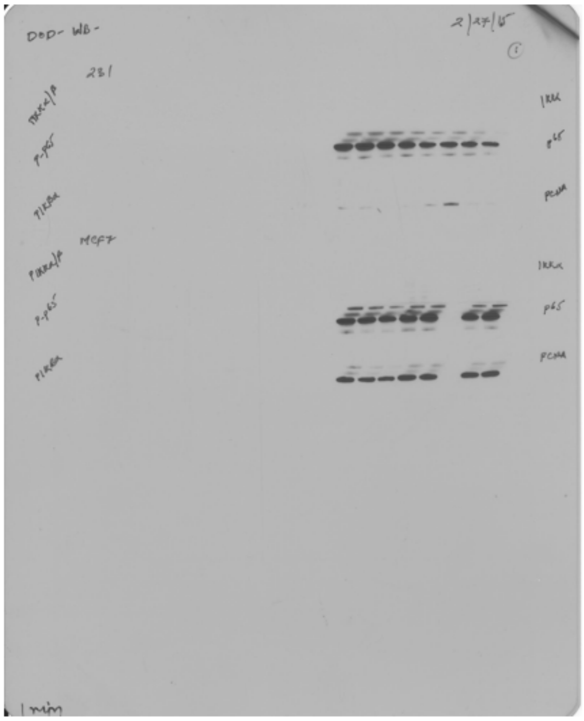
DOD-WB-
231
MCF7
1 min

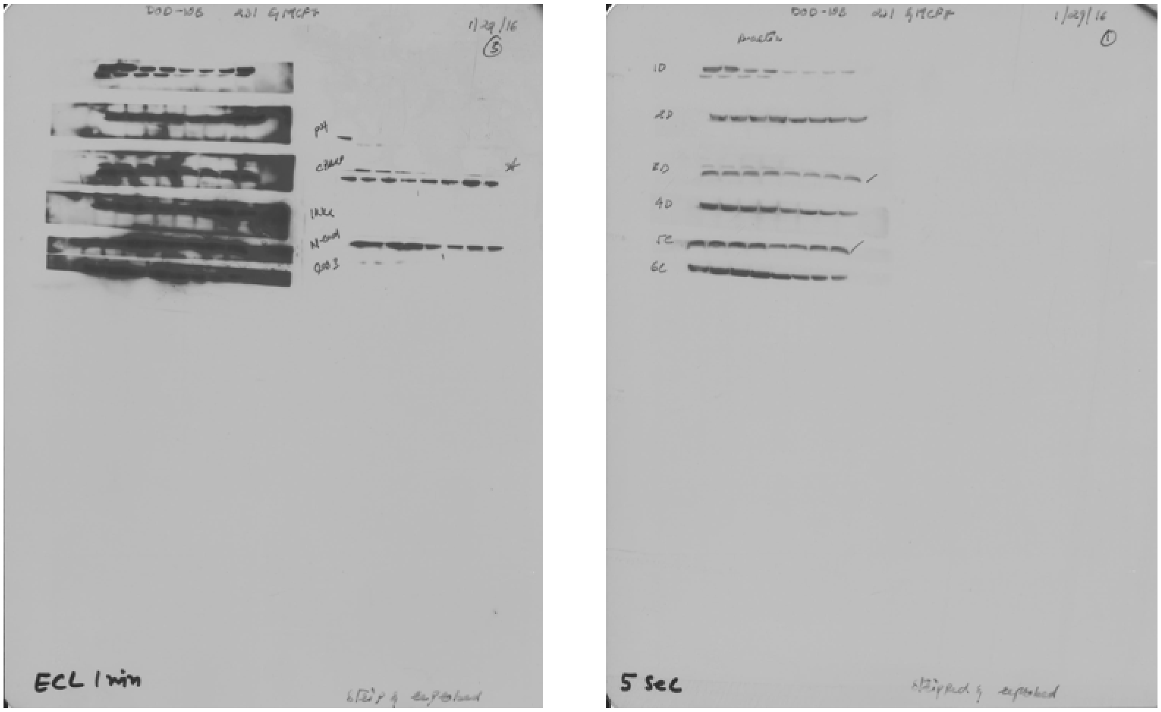

Figure S13. Original Western Blot of Figure 1d, 2e, and 3d.

Table S1. Reduction in Lymph node metastasis of MDA-MB-231 orthoxenografts in NOD-Scid mice following treatment with Anthos.

Group	Gross Examination for Macrometastasis				Histopathological Observations			
	Inguinal Lymph Nodes		Thoracic Lymph Nodes					
	Left	Right	Left	Right	Lymph nodes	Lung	Liver	Spleen
Control	++	++	+++	++	Metastasis	Normal	Normal	Normal
	+++	–	++	–	Metastasis	Metastasis	Normal	Normal
	+++	++	+++	+	Metastasis	Metastasis	Normal	Normal
	+++	+++	+++	+++	Metastasis	Normal	Normal	Normal
	++	+	++	+	Metastasis	Normal	Normal	Normal
	+++	–	+++	–	Metastasis	Metastasis	Normal	Normal
	++	–	–	–	Metastasis	Metastasis	Normal	Normal
	++	++	+++	++	Metastasis	Normal	Normal	Normal
	++	–	++	+	Metastasis	Normal	Normal	Normal
	++	+	++	–	Metastasis	Normal	Normal	Normal
Anthos (30 mg/kg)	+++	+	+++	+	Metastasis	Metastasis	Normal	Normal
	+++	+	++	++	Metastasis	Metastasis	Normal	Normal
	–	–	+	–	Metastasis	Normal	Normal	Normal
	–	–	–	–	Normal	Normal	Normal	Normal
	+++	–	++	–	Metastasis	Metastasis	Normal	Normal
	–	–	+	–	Metastasis	Normal	Normal	Normal
	+++	+	++	+	Metastasis	Metastasis	Normal	Normal
	–	–	–	–	Normal	Normal	Normal	Normal
Anthos (60 mg/kg)	–	–	–	–	Normal	Normal	Normal	Normal
	++	–	++	+	Metastasis	Metastasis	Normal	Normal
	+	–	+	–	Metastasis	Normal	Normal	Normal
	–	–	–	–	Metastasis	Normal	Normal	Normal
	+	–	–	–	Normal	Normal	Normal	Normal
	–	–	+	–	Normal	Normal	Normal	Normal

	+	–	++	+	Metastasis	Metastasis	Normal	Normal
	++	–	++	–	Metastasis	Normal	Normal	Normal
	+	+	+++	+	Metastasis	Metastasis	Normal	Normal

Gross examination of lymph nodes received a score of +, ++, and +++ which depicts the size of the enlarged lymph nodes. +, 0.1–0.5 mm; ++, 0.5–1 mm; and +++, >1 mm.
